# NMS-873 Leads to Dysfunctional Glycometabolism in A p97-Independent Manner in HCT116 Colon Cancer Cells

**DOI:** 10.3390/pharmaceutics14040764

**Published:** 2022-03-31

**Authors:** Shan Li, Feng Wang, Gang Zhang, Tsui-Fen Chou

**Affiliations:** 1 Division of Biology and Biological Engineering, California Institute of Technology, Pasadena, CA 91125, USA; fengwang@caltech.edu (F.W.); gzhang2@caltech.edu (G.Z.); 2 Proteome Exploration Laboratory, Beckman Institute, California Institute of Technology, Pasadena, CA 91125, USA

**Keywords:** p97 inhibitor, glycometabolism, resistance, protein stability, proteomics

## Abstract

Adenosine triphosphate (ATP)–competitive p97 inhibitor CB-5339, the successor of CB-5083, is being evaluated in Phase 1 clinical trials for anti-cancer therapy. Different modes-of-action p97 inhibitors such as allosteric inhibitors are useful to overcome drug-induced resistance, one of the major problems of targeted therapy. We previously demonstrated that allosteric p97 inhibitor NMS-873 can overcome CB-5083-induced resistance in HCT116. Here we employed chemical proteomics and drug-induced thermal proteome changes to identify drug targets, in combination with drug-resistant cell lines to dissect on- and off-target effects. We found that NMS-873 but not CB-5083 affected glycometabolism. By establishing NMS-873-resistant HCT116 cell lines and performing both cell-based and proteomic analysis, we confirmed that NMS-873 dysregulates glycometabolism in a p97-independent manner. We then used proteome integral solubility alteration with a temperature-based method (PISA T) to identify NDUFAF5 as one of the potential targets of NMS-873 in the mitochondrial complex I. We also demonstrated that glycolysis inhibitor 2-DG enhanced the anti-proliferative effect of NMS-873. The polypharmacology of NMS-873 can be advantageous for anti-cancer therapy for colon cancer.

## 1. Introduction

p97/VCP ATPase overexpresses in many cancers [1,2,3] and is a potential cancer therapy target [4,5,6,7]. In recent years, it has been reported that p97 inhibitors are being developed as anticancer candidates [8,9,10,11,12,13,14,15]. Among these research projects, ATP-competitive p97 inhibitor CB-5083 once entered a Phase 1 clinical trial, but unfortunately the trial was stopped due to an off-target effect on phosphodiesterase 6 (PDE6) [4]. The second-generation inhibitor CB-5339 has recently been reported [16], which can reduce the PDE6 side effect by fifteen-fold. CB-5339 is currently being evaluated in a Phase 1 clinical trial for acute myeloid leukemia and myelodysplastic syndrome (NCT04402541).

Although CB-5083 caused drug-induced resistance, several other types of p97 inhibitors have been reported as additional choices for cancer therapy that can overcome this challenge [14,17]. Among them, allosteric inhibitor NMS-873 showed good activity for both specific p97 inhibition and anti-proliferation [10,17], and thus could be considered a promising lead compound for further new p97 inhibitor development. Recent research reported that NMS-873 could inhibit mitochondrial oxidative phosphorylation [18]. However, the mechanism of action by which NMS-873 affects oxidative phosphorylation is not clear. Since p97 also affects mitochondrial and cellular metabolism functions [19,20], it is worth determining if NMS-873 inhibits oxidative phosphorylation by means that are p97-dependent or independent. Furthermore, deeper study of its mechanism of action and identifying its on- or off-target effects could be helpful for designing next-generation p97 inhibitors that overcome its off-target effects and provide candidate choice when other p97 inhibitors cause drug resistance.

The establishment of drug-induced resistant cell lines [21,22,23,24,25] has been used to study resistant mechanisms and to evaluate new drugs. These drug-induced resistant lines could also be considered as a suitable method to confirm on-target and off-target effects of a drug. First, we can examine the phenotype caused by compound treatment between parental and resistant lines through proper biochemistry or a cell-based approach. In addition, we can conduct high-throughput comparison of the dysregulated genes or proteins and their involved pathways through transcriptomic or proteomic profiling to identify resistance mechanisms and off-target drug effects. This knowledge will guide better therapeutic development.

To identify drug targets and mechanistic biomarkers, thermal proteome profiling (TPP), which combines cellular thermal shift assay (CETSA) and multiplexed quantitative mass spectrometry approaches, has been developed [26,27,28]. Recently, Gaetani et al. reported that a proteome integral solubility alteration with a temperature-based method (PISA T) [29], which can have decreased analysis time and sample consumption compared to traditional TPP, could be considered as a good choice for the initial screening of drug targets.

In this study, we established NMS-873-resistant cell lines and used tandem mass tag (TMT)-based quantitative proteomics and cell-based assays to conclude that NMS-873 dysregulates glycometabolism via a p97-independent manner. We obtained a list of possible NMS-873 binding targets through PISA T assay and identified NDUFAF5 as one of the potential binding targets of NMS-873 by overexpressing NDUFAF5 in cells to rescue the elevated level of lactate. We provided a valuable set of tools to further advance NMS-873 analogues as potential anti-cancer agents by targeting both p97 and mitochondria.

## 2. Material and Methods

### 2.1. Cell Lines and Reagents

Both HCT116 human colon cancer cells and 293T human embryonic kidney cells were purchased from ATCC (Manassas, VA, USA). HCT116 was maintained in RPMI1640 medium (Corning, Corning, NY, USA) supplemented with 10% fetal bovine serum (FBS, R&D Systems, Minneapolis, MN, USA) and 1% penicillin-streptomycin (P/S, Gibco, Waltham, MA, USA). 293T was maintained in DMEM medium (Corning) supplemented with 10% FBS and 1% P/S. Both cells were cultured at 37 °C in a 5% CO_2_ incubator (Thermo Fisher Scientific Inc., Waltham, MA, USA). Cells were routinely examined for mycoplasma using MycoAlert Mycoplasma Detection Kit (Lonza, Basel, Switzerland). Cells were plated at different densities, as described in the method of specified assays.

pMXs-NDUFAF5-HA plasmid, a gift from David Sabatini [30] (plasmid #86125, Addgene, Watertown, MA, USA), was transiently transfected into 293T cells with BioT reagent (Bioland, Paramount, CA, USA) to overexpress NDUFAF5-HA protein.

NMS-873-resistant cell lines (NMS-R1 and NMS-R2) were established, and mutation was identified by sequencing p97 cDNA according to published methods [17,31]. PCR and sequencing primers are listed in Table S8.

NMS-873, CB-5083, and ML240 were purchased from MedKoo Biosciences (Morrisville, NC, USA). UPCDC-30245 and 2-deoxy-d-glucose were purchased from Sigma-Aldrich (St. Louis, MO, USA). MG132 was purchased from Selleckchem (Houston, TX, USA). 2-Deoxy-d-glucose (Sigma-Aldrich, St. Louis, MO, USA) was dissolved in water, and all the other compounds were dissolved in dimethyl sulfoxide (DMSO) (Sigma-Aldrich, St. Louis, MO, USA) at appropriate stock concentrations and stored at −80 °C before future use in assays.

### 2.2. Lactate and Glucose Concentration Measurement

To examine compound treatment effects and compare NMS-873-resistant cell lines, HCT116 and NMS-R cells were seeded in 12-well plates (Corning, Corning, NY, USA) at a density of 8 × 10^5^/well in RPMI1640 medium with 5% of FBS and 1% of P/S. Twenty-four hours after seeding, the plates were changed with 1 mL of fresh medium per well, and either 5 μL of vehicle control or 200x compound stock was added and mixed well. Plates were incubated at 37 °C in a 5% CO_2_ incubator for six hours. The medium lactate and glucose concentrations were measured using a lactate analyzer (Lactate Scout+, SensLab GmbH, Leipzig, Germany) and glucose meter (AimStrip Plus, Germaine Labs, Indianapolis, IN, USA), respectively. After the test, cells were detached, and cell numbers were counted using the Countess II cell counter (Thermo Fisher Scientific Inc., Waltham, MA, USA). The concentrations were normalized to cell numbers to avoid seeding variations between wells.

To compare lactate levels with or without overexpressing NDUFAF5-HA, 293T cells were seeded in 24-well plates at a density of 1 × 10^5^/well in DMEM medium with 5% of FBS and 1% of P/S. The plasmid was transiently transfected into cells the next day. Forty-two hours later, the plates were changed with 0.5 mL of fresh medium per well, and either 2.5 μL of vehicle control or 200× compound stock was added and mixed well. Plates were incubated at 37 °C in a 5% CO_2_ incubator for six hours. The medium lactate concentrations were measured in the same way as above.

### 2.3. OCR and ECAR Measurement

HCT116 cells were seeded in the 8-well Seahorse XFp Cell Culture Miniplate (Agilent Technologies, Santa Clara, CA, USA) at a density of 1 × 10^4^/well in RPMI1640 medium containing 5% of FBS and 1% of P/S. Twenty-four hours later, plates were changed with fresh medium containing 5% DMSO or 2 μM NMS-873 and incubated for six hours. Then oxygen consumption rate (OCR) and extracellular acidification rate (ECAR) were determined using the Agilent Seahorse XFp Analyzer following the manufacturer’s instructions.

### 2.4. Cell Viability Measurement

HCT116 and NMS-R cells were plated in 384-well plates (Greiner Bio-One, Kremsmuenster, Austria) at a density of 750 cells per 30 μL per well in RPMI1640 medium containing 5% of FBS and 1% of P/S. Twenty-four hours after seeding, 8 μL medium containing DMSO (solvent control, final 1%) or serially diluted compound was added, and plates were incubated for another 48 h. Cellular viabilities were measured using Cell Titer Glo Luminescent Cell Viability Assay (Promega, Madison, WI, USA), and IC_50_ values were calculated using the percentage of growth of compound-treated wells vs. DMSO control.

### 2.5. Western Blot Assay

Cells were seeded in 6-well plates at a density of 1 × 10^6^/well. The next day, DMSO or compound was added and incubated for six hours. Cells were detached and harvested into 1.5 mL tubes. The pellets were lysed by adding 150 μL of lysis buffer (Thermo Fisher Scientific Inc., Waltham, MA, USA) containing 1% Triton X-100 and protease inhibitor, incubated on ice for 10 min with occasional vortex, then centrifuged (Eppendorf, Framingham, MA, USA) at 15,000 rpm for 10 min to separate the supernatant. Protein concentrations were measured with Bradford reagent (Bio-Rad, Hercules, CA, USA). After adding 4 × Laemmli sample buffer (Bio-Rad) containing 0.1 M DTT and heating at 95 °C for 5 min, 10 µg samples were loaded and separated with 4–20% Mini-PROTEAN TGX precast gels (Bio-Rad). Proteins were transferred to nitrocellulose membranes with the Trans-Blot Turbo system (Bio-Rad). Membranes were blocked for 30 min at room temperature and incubated with primary antibodies overnight at 4 °C, then washed and incubated with appropriate secondary antibodies for two hours at room temperature. Blots were imaged using ECL reagent (Millipore Sigma, Burlington, MA, USA) and ChemiDoc MP Imaging System (Bio-Rad).

### 2.6. TMT Labeling Proteomics

Cells were seeded and treated with the compound in the same method as described above, in the section “Western Blot Assay”. Cell pellets were prepared for mass spectrometry acquisition by following the EasyPep Mini MS Sample Prep Kit (Thermo Fisher Scientific Inc.). Peptide concentrations were determined with the Quantitative Fluorometric Peptide Assay (Thermo Fisher Scientific Inc.).

A total of 15 μg of peptide was prepared to label with TMTpro 16 plex reagents (Thermo Fisher Scientific Inc.) according to the manufacturer’s instructions. Labeled samples were combined and dried with vacuum centrifugation. Samples were then separated into eight fractions using the High pH Reversed-Phase Peptide Fractionation Kit (Thermo Fisher Scientific Inc.). The fractions were dissolved in 0.1% formic acid, and peptide concentrations were determined with Quantitative Colorimetric Peptide Assay (Thermo Fisher Scientific Inc.).

TMT labeled sample LC-MS/MS acquisitions were performed using an EASY-nLC 1000 connected to an Orbitrap Eclipse Tribrid mass spectrometer (Thermo Fisher Scientific Inc.). An amount of 1 μg of each fraction was loaded on an Aurora UHPLC Column (Ionopticks, Fitzroy, Australia) and separated with a 136-min method, as described previously [14]. MS1 scans were acquired in the Orbitrap at 120 k resolution with a scan range of 350–1600 *m*/*z*. The AGC target was 8 × 10^5^, and the maximum injection time was 50 ms. MS2 scans were acquired with collision-induced dissociation (CID) activation type with the Iontrap. The isolation window was 0.4 *m*/*z*, collision energy was 35%, maximum injection time was 45 ms, and AGC target was 10^4^. MS3 scans were acquired with higher-energy collisional dissociation (HCD) activation type in the Orbitrap at 50 k resolution with a scan range of 100–500 *m*/*z*. The isolation window was 0.7 *m*/*z*, collision energy was 55%, maximum injection time was 86 ms, and AGC target was 2.5 × 10^5^. System control and data collection were performed with Xcalibur software (Thermo Fisher Scientific Inc.).

### 2.7. PISA T Assay

HCT116 cells were harvested from one 80% confluent 15 cm plate. Cell pellets were lysed with 1 mL lysis buffer (DPBS containing protease inhibitor and 0.2% DDM) and centrifuged at 16,000× *g* for 10 min at 4 °C. The supernatant was carefully transferred to a new tube, and protein concentration was measured with Bradford reagent. Protein concentration was diluted to 2 μg/μL with lysis buffer to reach a final concentration of 0.5% DMSO or 50 μM NMS-873, and the treated supernatants were incubated at room temperature for 30 min. Each group was then aliquoted into 2 × 8 PCR tubes (replicates) and treated at eight temperature points (45, 48, 51, 54, 57, 60, 63, and 66 °C); at each of these temperature points, the tubes were heated for three minutes using the thermal cycler (Bio-Rad) and then left at room temperature for another three minutes. Equal volumes of samples treated at 45–54 °C and 57–66 °C temperature ranges, respectively, were combined. The combinations were centrifuged at 20,000× *g* for 20 min at 4 °C. Protein concentration was measured with Bradford reagent; 40 μg of proteins were transferred to a new tube, and water and 5% SDS were added to get 100 μL samples containing 1% SDS. Next, 2 µL of 500 mM TCEP and 5 µL of 500 mM fresh prepared CAA were added, and samples were incubated at 95 °C for 10 min. Next, six volumes of pre-chilled acetone were added to precipitate the protein, and samples were stored at −20 °C overnight. Precipitated protein was collected by centrifuging samples at 20,000× *g* for 10 min at 4 °C. The dry 40 µg pellets were resuspended with 40 µL of 100 mM TEAB buffer containing 0.4 µg Lys-C (Wako Chemicals, Osaka, Japan) and 0.8 µg Trypsin (Thermo Fisher Scientific Inc.). The sample was then digested at 37 °C overnight. Peptide concentrations were determined with Quantitative Fluorometric Peptide Assay (Thermo Fisher Scientific Inc.). 15 μg of the peptide was used to prepare the mass spectrometry sample, as described earlier in the section “TMT Labeling Proteomics”. We made two sets of independent TMT labeling and mass spectrometry acquisition and analysis data by combining the two sets as experimental replicates.

### 2.8. In Silico Docking Analysis of NMS-873 with NDUFAF5

The protein sequence of human NDUFAF5 was retrieved from UniProt and modeled using I-TASSER, due to the unavailability of a template with sufficient homology [32]. The structural quality of the model was assessed by MolProbity for the Ramachandran Plot [33] and ProSA [34]. A molecular dynamic simulation for the NDUFAF5 model was carried out with water as the solvent for 10,000 picoseconds using GROMACS under default parameters [35]. The PDB protein and molecules were prepared by adding hydrogen and were converted to PDBQT format by Open Babel [36]. A grid box with dimensions of 40 × 40 × 40 Å (41.89, 37.08, 34.245) with a spacing of 0.375 Å was constructed around the docking area using Autodock 4.2 software (Scripps Research, San Diego, CA, USA) [37]. Molecules were docked using Vina with exhaustiveness grade 8, with up to nine poses saved per molecule. The docking procedure was carried out for the unchanged conformation of the receptor and flexible ligand molecules. The lowest-energy conformations were selected, and the interactions between the ligand and NDUFAF5 were analyzed. Accelrys Discovery Studio Visualizer 4.0 (Accelrys, San Diego, CA, USA) was used for interaction visualization.

### 2.9. Quantification and Statistical Analysis

Proteomic analyses were performed with Proteome Discoverer 2.4 (Thermo Fisher Scientific Inc.) using the Uniprot human database and the SequestHT with Percolator validation. TMTpro (Any N-Terminus) was set as a static N-Terminal Modification; TMTpro (K) and carbamidomethyl (C) were set as static modifications; oxidation (M) was set as a dynamic modification; acetyl (protein N-term), Met-loss (Protein N-term M), and Met-loss + acetyl (Protein N-term M) were set as dynamic N-Terminal modifications. Normalization was performed relative to the total peptide amount. Further analyses were performed using the normalized abundance as below: limma analyses were performed using R studio following the user guide [38]; PCA analyses were generated with PD2.4 and plotted using Prism 8; volcano plots and heatmaps were generated with Prism 8; Venn plots were generated with FunRich_3.1.3; enrichment analyses were performed using g:Profiler [39]; bubble plots were generated with Origin 2019b (OriginLab Corporation, Northampton, MA, USA); and the protein–protein interaction network was performed using the STRING database [40].

Other statistical analyses were carried out by multiple *t*-tests or non-paired one-way ANOVA using Prism 8. *p*-values less than 0.05 were considered as statistically significant; * *p* < 0.05, ** p < 0.01, *** *p* < 0.0001.

## 3. Results

### 3.1. An Allosteric p97 Inhibitor, NMS-873, Regulates Glycometabolism

**Figure 1:**
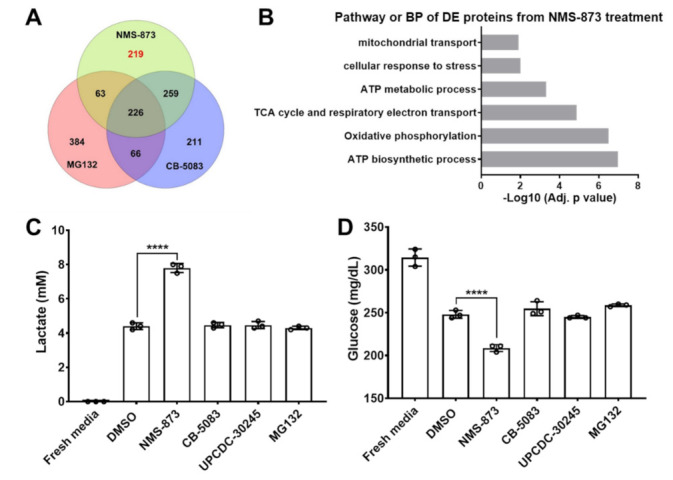
Only NMS-873 promotes glycolysis and inhibits oxidative phosphorylation. (**A**,**B**) Using TMT-based quantitative proteomics to distinguish the unique pathway of NMS-873 [30]. Venn diagram of DE proteins identified from HCT116 cells at 6 h-treatment with MG132, CB-5083, and NMS-873. DE proteins were defined as treatment vs. DMSO control, *p* < 0.05; *n* = 2 (**A**). The representative KEGG pathway or GO biological process from a functional enrichment analysis of the 219 DE proteins identified only in the NMS-873 treatment group (**B**). (**C**,**D**) Lactate (**C**) and glucose (**D**) concentration in the cultured medium were measured after 6 h incubation with DMSO, 4 μM NMS-873, 4 μM CB-5083, 4 μM UPCDC-30245, or 1 μM MG132. Statistical analyses were carried out by non-paired one-way ANOVA (treatment vs. DMSO) using Prism 8. *p*-values are shown as **** *p* < 0.0001. All of the data from (**C**,**D**) are presented as mean ± SD from triplicate experiments.

We recently used proteomic analysis of the HCT116 colon cancer cell line to define the cellular and molecular responses upon proteasome and p97 inhibition [41]. We previously focused on the proteins that are changed by both CB-5083 and NMS-873 [41]. Here, we focus on the proteins that are uniquely affected by NMS-873 since there is an ongoing effort to develop NMS-873 analogues. To do so, we started by overlapping differentially expressed (DE) proteins from the treatment of MG132 (a proteasome inhibitor), CB-5083, and NMS-873, and found that 219 proteins are specifically dysregulated only by NMS-873 after 6 h treatment (Figure 1A, Table S1, Supplementary Materials). Functional enrichment analysis of these 219 DE proteins uniquely dysregulated by NMS-873 identified the top relevant changed biological processes and pathways, presented in Figure 1B. The analysis showed that these DE proteins are primarily involved in ATP biosynthetic and metabolic processes, oxidative phosphorylation, TCA cycle and respiratory electron transport, and mitochondrial transport, which indicates that NMS-873 may affect glycometabolism.

Recently, Bouwer et al. observed media acidification when treating human renal tubule cells and mouse fibroblasts with NMS-873 and verified that NMS-873 inhibits mitochondrial oxidative phosphorylation by inhibiting complex I potently and complex V weakly [18]. We also consistently observed that the medium of HCT116 cells turned yellow when treated with NMS-873 at 6 h and is more apparent at 24 h (Figure S1A, Supplementary Materials), whereas wells with MG132 or other p97 inhibitors, CB-5083 and UPCDC-30245 (another allosteric p97 inhibitor) [12,42], remained pink like the DMSO control. Promotion of medium acidification suggested that NMS-873 might induce glycolysis. The cell viability after 6 h treatment with NMS-873 does not change (Figure S1B, Supplementary Materials). Next, we simultaneously measured the real-time oxygen consumption rate (OCR) and extracellular acidification rate (ECAR) for HCT116 cells following six hours of treatment with either DMSO or 2 μM of NMS-873. As shown in Figure S1C (Supplementary Materials), the OCR of the NMS-873 treated group was lower than that of DMSO control, which confirms that NMS-873 inhibited mitochondrial oxidative phosphorylation. Figure S1D (Supplementary Materials) shows that the ECAR of the NMS-873 group was higher than that of the DMSO group, suggesting that NMS-873 promoted glycolysis.

We measured the lactate and glucose concentrations in the HCT116 cell culture medium following six hours of treatment with DMSO, 4 μM of NMS-873, 1 μM of MG132, 4 μM of CB-5083, or 4 μM of UPCDC-30245. Figure 1C shows that, compared to the DMSO control, the lactate level is higher in the NMS-873 treatment group. Figure 1D shows that the medium glucose concentration of the NMS-873 treatment group is lower than that of the DMSO control. These results concluded that NMS-873 treatment promotes medium glucose consumption and lactate production. Conversely, neither MG132 nor other p97 inhibitors showed any significant effect on the medium glucose and lactate levels. Combined with the enrichment analysis results and cell culture phenotypes, these results suggest that only NMS-873 affected glycometabolism.

### 3.2. 2-DG Blocks NMS-873-Induced Glycolysis and Enhances Its Anti-Proliferative Activity

**Figure 2:**
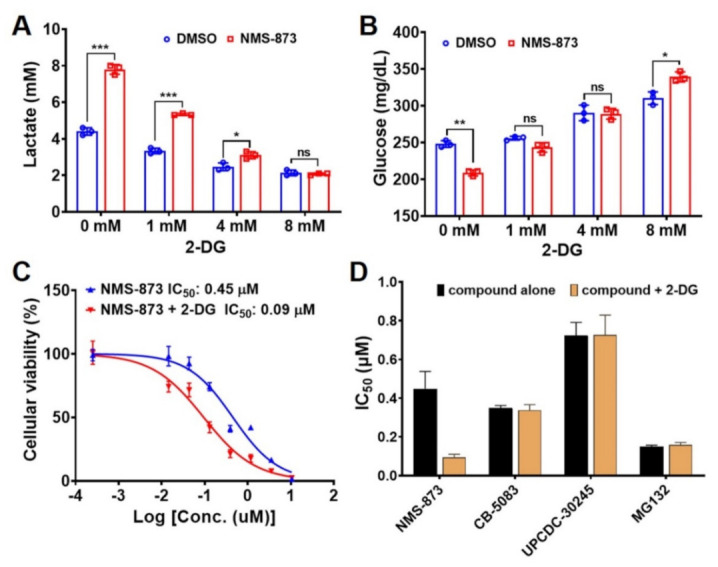
Synergistic anti-proliferative effect of 2-DG and NMS-873 in HCT116. (**A**,**B**) 2-DG inhibited NMS-873-induced glycolysis. Lactate production (**A**) and glucose concentration (**B**) were measured in the medium of HCT116 cells after different concentrations of 2-DG co-cultured with DMSO or 4 μM NMS-873 for 6 h. Data are presented as mean ± SD from triplicate experiments. Statistical analyses were carried out by multiple *t*-tests (NMS-873 vs. DMSO) using Prism 8. *p*-values are shown as * *p* < 0.05, ** *p* < 0.01, *** *p* < 0.001 and ns stands for non-significant difference. (**C**,**D**) Anti-proliferation activities of the compound alone treated and co-treated with 1 mM 2-DG against HCT116 cells after 48 h treatment. The cell viability of 1 mM 2-DG was ~73% compared with DMSO (Figure S3C, Supplementary Materials). The IC_50_ of compounds in co-treatment was calculated by normalizing to the single treatment of 1 mM 2-DG. Data are presented as mean ± SD from quadruplicate experiments.

To evaluate the glycolysis effect caused by NMS-873, we used an inhibitor of glycolysis, 2-deoxy-d-glucose (2-DG). 2-DG competes with the cellular uptake of glucose by glucose transporter [43] and can be phosphorylated to 2-deoxy-d-glucose-6-phosphate (2-DG-6-P). However, it cannot be further metabolized and thus leads to 2-DG-6-P accumulation and glycolysis inhibition [44]. In the presence of increased 2-DG concentrations, the NMS-873 induced production of lactate and reduction of glucose was blocked (Figure 2A,B). Specifically, at 8 mM of 2-DG, the lactate level displayed no difference between DMSO and NMS-873 (Figure 2A), whereas the medium glucose level was higher in the NMS-873 treated group than that of DMSO group (Figure 2B). These results suggest that 2-DG inhibited NMS-873-induced glycolysis. In addition, NMS-873 not only induced glycolysis but also inhibited glucose consumption via other cellular processes.

Due to many chemotherapy drugs affecting the metabolic process, the combination of a cytotoxic drug with an antimetabolite has been proposed as a promising approach for enhancing anticancer efficiency and overcoming resistance caused by a switch of metabolic pathways [45,46]. 2-DG, as a glycolysis inhibitor, has been explored as an adjuvant agent to sensitize various cancer cells to clinically used anticancer drugs [44,47,48,49]. We thus measured the cellular viabilities of HCT116 by treating with NMS-873 alone or co-treating with 1 mM of 2-DG. As shown in Figure 2C, the IC_50_ of NMS-873 was five times that in co-treatment, indicating that a combination of 2-DG and NMS-873 exhibited an enhanced anti-proliferative activity. We then determined if this is an additive or synergetic effect by calculating the coefficient of drug interaction (CDI) values to be less than 1, which indicates that the drugs are synergistic (Figure S3E, Supplementary Materials) [50,51,52,53]. In contrast, the presence of 2-DG did not alter the IC_50_ of CB-5083, UPCDC-30245, and MG132 (Figure 2D). These results indicate that the enhanced effect by 2-DG is unique to NMS-873 rather than an effect of disturbing UPS by p97 or proteasome inhibition. They are consistent with the fact that when oxidative phosphorylation is inhibited by NMS-873, inhibition of glycolysis simultaneously by 2-DG will lead to increased cancer cell death due to both ATP production pathways being blocked. This effect is similar to the synergistic effect of phenformin and oxamate [54].

### 3.3. NMS-873 Interfered with Glycometabolism in a p97-Independent Manner

To study whether NMS-873 leads to dysfunctional glycometabolism because it inhibits p97 in a unique way that is different from other p97 inhibitors tested here, we established NMS-873-resistant HCT116 cell lines using methods described previously [17,31,55]. A total of six resistant clones were isolated and expanded. By cloning and sequencing p97 cDNA from these resistant clones, we identified five clones containing a heterozygous A530T p97 mutant, consistent with the previous study [56]. We also got a clone that is a homozygous A530T p97 mutant (Figure S2B, Supplementary Materials). All five heterozygous A530T clones showed similar increase in IC_50_ values using an anti-proliferation assay (Figure S2A, Supplementary Materials). We therefore chose one of the heterozygous A530T clones (termed NMS-R1) and the homozygous A530T clone (termed NMS-R2) for further study (Figure S2B, Supplementary Materials).

**Figure 3:**
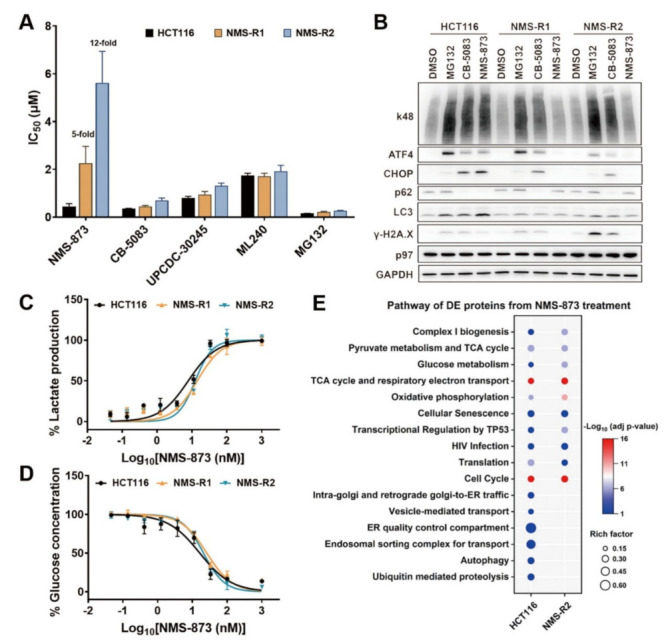
NMS-873 affects glycometabolism in a p97 independent manner. (**A**) Anti-proliferation activity of p97 inhibitors and MG132 against HCT116 and NMS-R cells after 48 h treatment. Data are presented as mean ± SD from quadruplicate experiments. (**B**) HCT116 and NMS-R cell lines were treated with 1 μM MG132, 2 μM CB-5083, or 4 μM NMS-873 for 6 h and analyzed by western blot for UPR (k48, ATF4, CHOP), autophagy (p62, LC3), and apoptosis (γ-H2A.X) biomarkers of p97 inhibition. (**C**,**D**) Lactate production (**C**) and glucose consumption (**D**) were measured in the medium of parental and NMS-873-resistant cells after being treated with different concentrations of NMS-873 for 6 h. Plots are presented as mean ± SD from triplicate experiments. (**E**) Bubble plot comparing related pathways of the DE proteins from proteomic analysis of 4 μM NMS-873-treated HCT116 and NMS-R2 cell lines (*n* = 4).

We compared the anti-proliferative activities of NMS-873, MG132, and three other p97 inhibitors in both parental and NMS-873-resistant HCT116 cell lines. As shown in Figure 3A, NMS-R1 and NMS-R2 are 5-fold and 12-fold less sensitive to NMS-873 than parental HCT116. In contrast, both MG132 and other p97 inhibitors inhibited the growth of HCT116 and NMS-873-resistant cells with similar or slightly increased (<2-fold) IC_50_ values. We next examined the known p97 biomarkers after six hours of treatment with 1 μM of MG132, 2 μM of CB-5083, and 4 μM of NMS-873 on parental and NMS-R HCT116 cell lines (Figure 3B and Figure S2C–E). Similar changes of UPR (k48, ATF4, CHOP), autophagy (p62, LC3), and apoptosis (γ-H2A.X) biomarkers were observed with MG132 and CB-5083 treatment for all three cell lines; these changes are consistent with previous studies and these results indicate that the NMS-R HCT116 has functional UPR and proteasome substrates are accumulated by MG132 and CB-5083 [17,46]. Conversely, NMS-873 led to changes in parental HCT116 but not in the NMS-R HCT116 cells, which is also consistent with the previous report [56]. Taken together, these data suggest that NMS-873 resistance in NMS-R HCT116 cell lines was driven by p97 A530T mutation.

To evaluate the glucose and lactate levels in the A530T p97 mutant lines, we treated HCT116 and NMS-R cell lines with different concentrations of NMS-873 for six hours. We observed that lactate concentration increased similarly in a concentration-dependent manner in both WT and A530T p97 HCT116 lines (Figure 3C and Figure S3A, Supplementary Materials) and glucose decreased similarly (Figure 3D and Figure S3B, Supplementary Materials). As shown in Figure 3B, there is no p97-inhibition biomarker response, even at 4000 nM of NMS-873 treatment on NMS-R cell lines. Additionally, the synergistic effect of co-treatment with NMS-873 and 2-DG was also found in NMS-873-resistant cell lines (Figure S3D,E; Supplementary Materials). Taken together, these results indicated that NMS-873 induced glycolysis in a p97-independent manner.

### 3.4. Quantitative Proteomics to Identify p97 Dependence of the NMS-873 Affected Pathways

To fully understand the p97-dependent and independent effects caused by NMS-873, we used TMT-based quantitative proteomics to compare the differential effects of NMS-873 treatment in HCT116 and NMS-R2 cell lines. Each cell line was treated with DMSO or 4 μM NMS-873 for six hours in four replicates. A total of 8007 proteins were identified, and 7828 were quantified across all 16 samples (Table S2, Supplementary Materials). Principal component analysis (PCA) showed that each group was well separated from others, except for one outlier from the DMSO-treated NMS-R2 cell line (Figure S4A, Supplementary Materials). Therefore, we excluded that sample and used the other 15 samples for further analysis. Through differential expression analysis using limma (Table S2, Supplementary Materials), we identified 2028 proteins with significantly different quantities (*p* < 0.05) between NMS-873- and DMSO-treated HCT116 cells (Figure S4B, Supplementary Materials), and 2212 proteins between NMS-873- and DMSO-treated NMS-R2 cells (Figure S4C, Supplementary Materials). Subsequently, the DE proteins from each cell were uploaded separately to g:Profiler to perform a functional enrichment analysis. By comparing significantly enriched KEGG and Reactome functional pathways from two cells (Figure 3E), we observed that some well-known p97-related functions, such as ubiquitin-mediated proteolysis, autophagy, ER quality control, and Golgi-to-ER traffic, as well as endosomal and vesicle-associated transport, were only identified in parental HCT116 cells. However, glycometabolism-related pathways, such as oxidative phosphorylation, TCA cycle, glucose metabolism, and complex I biogenesis, were affected by NMS-873 treatment in both HCT116 and NMS-R2 cells. These results suggested that NMS-873 could not inhibit p97 to regulate p97-associated functions in the resistant cell line, but it could still affect glycometabolism because it functions independently of p97.

### 3.5. NMS-873 Affects the Thermal Stability of Mitochondrial Complex I and ATP Synthase

**Figure 4:**
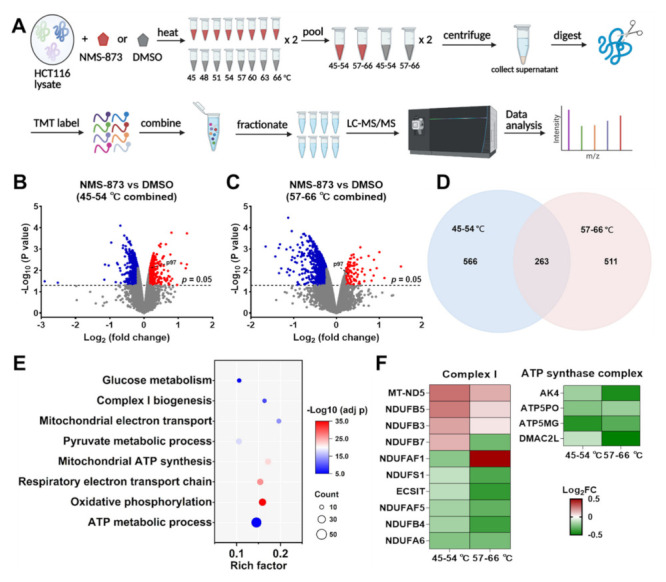
Using thermal proteome profiling to identify potential NMS-873 targets**.** (**A**) Workflow of the PISA T assay used in this study. (**B**,**C**) Volcano plots presenting DS proteins at two temperature ranges; DS proteins were defined as treatment vs. DMSO control, *p* < 0.05 and │logFC│≥ logFC of p97. (**D**) Venn plots overlapping DS proteins from different temperature ranges to get the list of all possible NMS-873 binding targets. (**E**) Bubble plot showing glycometabolism-associated pathways and GO biological processes from functional enrichment analysis. See also Table S5 (Supplementary Materials). (**F**) Heatmap listing fold change of DS proteins classified to mitochondrial complex I or ATP synthase complex. See also Table S6 (Supplementary Materials).

Since the fact that NMS-873 dysregulates glycometabolism is independent of p97, we sought to identify how NMS-873 affects glycometabolism. The proteome-wide cellular thermal shift assay (CETSA) is used to determine protein interaction changes in the presence of drug [57,58]. We chose to use a PISA T assay [29] to identify possible NMS-873 targets by comparing protein thermal stability under different conditions. We first used crude cell extracts prepared by 4 freeze-thaw cycles to perform PISA T, in which samples of the whole temperature range 45–60 °C (*n* = 3) were combined. TMT-labeled proteomics identified 7409 proteins and quantified 6855 proteins (Table S3, Supplementary Materials). 570 proteins were identified as differentially stabilized (DS) proteins by comparing NMS-873-treated samples with DMSO-treated samples (*p* < 0.05, Figure S5 and Table S3, Supplementary Materials). Unfortunately, we did not identify p97 as one of the targets. To improve from this analysis, we found a previous study suggested that the selection of heating temperature points could affect the PISA T assay’s sensitivity [59]. We changed our method to prepare cell lysates with 0.02% n-dodeyl-β-d-maltoside (DDM) and separated two temperature groups as shown in the modified workflow of the PISA T assay (Figure 4A). From the combined analysis of two independent sets of TMT-labeled quantitative proteomics, 7996 proteins were identified and 6196 proteins were quantified (Table S4, Supplementary Materials). PCA analysis showed that biological replicates had close principal component scores (Figure S6A, Supplementary Materials), indicating that they are correlated well with each other. The combined temperature ranges contributed to the most changes, and compound treatment contributed to the second-most changes in protein thermal stability. From differential expression based on limma analysis, we found p97 was thermally stabilized by NMS-873 (Figure 4B,C). By using the fold change of p97 as a cutoff (*p* < 0.05, |logFC| ≥ logFC of p97), 829 and 774 DS proteins were identified, respectively, in 45–54 °C and 57–66 °C samples (Figure 4B,C). By overlapping DE proteins from two temperature ranges (Figure 4D), a total of 1340 possible NMS-873 binding targets were identified (Table S4, Supplementary Materials); this list could be a valuable reference for future research using NMS-873.

To find targets associated with glycometabolism, we performed functional enrichment analysis on the DS proteins and identified some glycometabolism-related biological processes (Figure 4E). Protein–protein interaction (PPI) analysis of DS proteins listed in these glycometabolism-related enrichment processes was further carried out using STRING (Figure S6B, Supplementary Materials). Among them, four DE proteins of the ATP synthase complex and ten DE proteins from complex I were identified (Figure 4F, Table S6, Supplementary Materials). These data are consistent with the previous report [18] that NMS-873 is a potent complex I inhibitor and a weak ATP synthase inhibitor. Overall, our data suggest that NMS-873 could affect the stability of complex I and ATP synthase to inhibit their activities, as shown recently [18].

### 3.6. Overexpression of NDUFAF5 Rescues NMS-873 Induced Lactate Production

**Figure 5:**
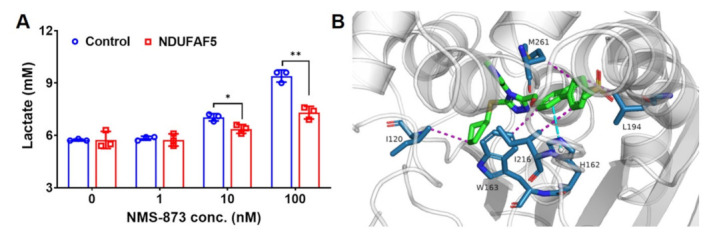
Overexpression of NDUFAF5 rescues NMS-873 induced lactate production. (**A**) Lactate concentrations in a medium of 293T cells were measured with or without overexpression of NDUFAF5-HA and treated with DMSO or NMS-873 for 6 h. Data are presented as mean ± SD from triplicate experiments. Statistical analyses were carried out by multiple *t*-tests (NDUFAF5 vs. control) using Prism 8. *p*-values are shown as * *p* < 0.05 and ** *p* < 0.01. (**B**) Docking study of NMS-873 with NDUFAF5. NMS-873 is shown as green sticks. The residues at the binding site are shown as teal sticks. The hydrophobic interaction and the π-π stacking are shown as purple and cyan dotted lines, respectively.

Since NMS-873 mainly affects protein thermal stability and activity [18] of complex I, we next tried to identify the protein targets of NMS-873 in complex I. By overlapping our first PISA T assay using crude cell extracts with the modified PISA T assay result, we found an overlap of 73 protein in both PISA T assays (Table S7, Supplementary Materials). Among them, NDUFAF5 was commonly down-regulated and showed a similar log_2_FC in both PISA T assays (Figure 4F and Figure S5, Supplementary Materials). This led us to overexpress NDUFAF5 in cells and then examined the phenotype of NMS-873 treatment. We transiently transfected the NDUFAF5-HA plasmid into 293T cells for better expression of NDUFAF5-HA [30] (Figure S6C, Supplementary Materials). Specifically, 42 h after transfection, the medium was changed to a fresh DMEM medium containing DMSO or NMS-873 and incubated for an additional six hours. We demonstrated that overexpression of NDUFAF5-HA could significantly inhibit NMS-873-induced lactate production (Figure 5A).

To further explore the potential binding mode of NMS-873 with NDUFAF5, a docking study of NMS-873 was performed (Figure 5B). There were multiple hydrophobic interactions between the residues and NMS-873, such as I120, W163, L194, I216, and M261. In addition, the imidazole ring of H162 interacted with the phenyl ring in the middle of NMS-873 by π–π stacking. All these interactions, including hydrophobic and π–π stacking, suggest that NMS-873 may bind with NDUFAF5. These data suggest that NMS-873 may bind with NDUFAF5 to inhibit complex I activity, and therefore, overexpression of NDUFAF5-HA prevents NMS-873 binding to endogenous complex I, thus affecting NMS-873 to inhibit oxidative phosphorylation and promote glycolysis.

## 4. Discussion

p97/VCP overexpresses in many cancers [1,2,3] due to its essential role in protein homeostasis and protein quality control, and is a promising anticancer target [4,5,6,7]. CB-5339 [16], the second-generation ATP-competitive p97 inhibitor, could overcome the PDE6 side effect caused by CB-5083, and is being evaluated in two clinical trial studies. Cell lines that are resistant to CB-5083 because of induced p97-resistant mutations have been reported [11,17]. To overcome CB-5083/CB-5339 resistance, it is critical to develop new ATP-competitive inhibitors that bind to a different site of p97, or other types of p97 inhibitors such as covalent and allosteric inhibitors. Among the reported inhibitors, NMS-873 showed excellent specific p97 inhibition and cell toxicity activity [10] and has been proven to overcome CB-5083-induced resistance [17]. Thus NMS-873 is a promising candidate for p97 inhibitor development. A recent study reported that NMS-873 could inhibit oxidative phosphorylation [18]. In our proteomic comparison of different p97 inhibitors and MG132 [41], we found that only NMS-873 affects the TCA cycle and oxidative phosphorylation (Figure 1A,B). We also examined the medium lactate and glucose concentrations from different compound-treated HCT116, and found that only NMS-873 promotes medium glucose consumption and lactate production (Figure 1C,D). Considering that p97 also affects mitochondrial function [19,20], it is necessary to study whether NMS-873 affects glycometabolism via p97 inhibition.

In this study, we established NMS-873-induced resistant cell lines harboring the A530T mutant of p97 (Figure S2B, Supplementary Materials), which has been reported to cause a five-fold decrease in the p97 inhibition potency of NMS-873 [56]. We used a cell viability assay that confirmed the anti-proliferation resistance of NMS-873 on NMS-R cell lines (Figure 3A). We also detected p97-associated biomarkers by western blot and demonstrated that NMS-873 lost p97 inhibition at 4 μM and six hours of treatment on NMS-R cells (Figure 3B and Figure S2C–E, Supplementary Materials). Furthermore, we compared cellular pathways affected by NMS-873 treatment on HCT116 and NMS-R2 by TMT-based quantitative proteomics. The functional enrichment analysis result (Figure 3E) showed that NMS-873 could not affect p97-related pathways in NMS-R2 but still could affect glycometabolism-associated functions. This indicates that NMS-873 leads to dysfunctional glycometabolism by means that are independent of p97. The enrichment data also showed that NMS-873 affects some other pathways in HCT116 and NMS-R2, suggesting that NMS-873 may also affect these cellular functions through p97-independent manners. We compared medium lactate and glucose concentrations of HCT116 and NMS-R lines to confirm the independent dysfunctional glycometabolism effect. All the three cell lines showed similar dose-dependent lactate increasing and glucose decreasing by NMS-873 treatment, and the IC_50_s are ~10 nM (Figure 3C,D). A previous study reported that NMS-873 inhibits complex I with an IC_50_ of 1.3 μM in L939 normal fibroblast cells [18], 100 times higher than our result in HCT116 colon cancer cells. This observation indicates that NMS-873 may work more efficiently to inhibit oxidative phosphorylation on tumor cells.

To investigate how NMS-873 affects glycometabolism, we used a PISA T assay, an optimized TPP approach, to identify NMS-873 binding targets (Figure 4A). By overlapping DS proteins from the two combined temperature ranges, we derived a list of all possible proteins directly affected by NMS-873 by using fold change of p97 as cutoff (Table S4, Supplementary Materials), which could be a valuable reference data resource for future research on the mechanism of action of NMS-873. We used functional enrichment analysis to narrow down the DS proteins associated with glycometabolism (Figure 4E) and used STRING PPI analysis (Figure S6B, Supplementary Materials) to comprehend the network relationship. From these analyses, we found that the thermal stabilities of 17 complex I subunit proteins and five proteins belonging to ATP synthase were affected by NMS-873 treatment (Figure 4F). This suggests that NMS-873 could directly affect the stabilities of mitochondrial complex I and ATP synthase.

In addition, we found that NDUFAF5, a complex I assembly factor, showed similar log_2_FC in a both PISA T assays (Figure 4 and Figure S5, Supplementary Materials). We therefore chose to see the effect of this protein in 293T cells, we observed that the overexpression of NDUFAF5-HA reversed the lactate production induced by NMS-873 treatment (Figure 5A), indicating that overexpressed NDUFAF5 can potentially block binding of NMS-873 to inhibitor complex I. In addition, we used a docking study to explore the binding mode of NMS-873 with NDUFAF5 (Figure 5B), and the interactions suggested that NMS-873 can possibly bind to NDUFAF5”.

**Figure 6:**
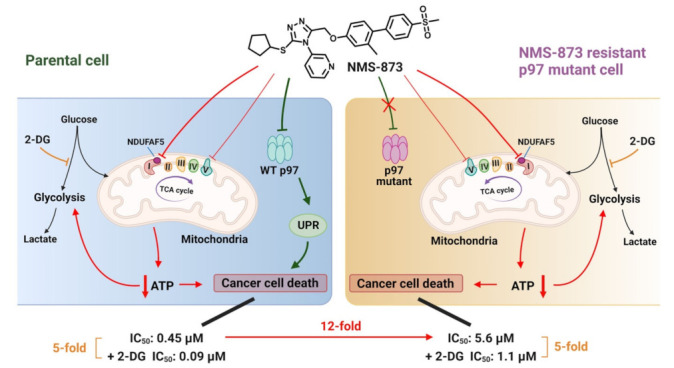
Schematic of NMS-873′s polypharmacology effects. Using chemical proteomics with parental and NMS-873 resistant HCT116 cells, we found that NMS-873 induces cancer cell death via blocking p97 (leads to UPR, green color) and mitochondrial functions (leads to decreased cellular ATP level, red color). NMS-873 induced glycometabolism dysregulation via a p97-independent manner. Using thermal proteome profiling, we discovered NMS-873 affects the stability of two mitochondria protein complexes and among them, NDUFAF5 was significantly down-regulated. Overexpression of NDUFAF5 blocks NMS-873-induced glycometabolism dysregulation and a silico docking study predicts NMS-873 can bind to NDUFAF5. Our findings lead us to propose that the polypharmacology effects of NMS-873 targeting both p97 and NDUFAF5 explains why there is only a 12-fold increase in IC_50_ for anti-proliferative activity in NMS-873-resistant p97 mutant cancer cells (CB-5083-resistant p97 mutant cancer cells is ~100-fold resistant [17]) and why the glycolysis inhibitor 2-DG has a synergistic anti-proliferative effect with NMS-873 in both parental and mutant cells (5-fold increase in anti-proliferative activity).

Moreover, we also examined the co-treatment of glycolysis inhibitor 2-DG and NMS-873 in this study. We found that 2-DG could inhibit NMS-873-induced glycolysis (Figure 2A,B) and cause a five-fold increase of the anti-proliferative effect of NMS-873 in both HCT116 and NMS-R cell lines (Figure 2C and Figure S3D,E). The coefficient of drug interaction (CDI) values of are all less than 1 in both HCT116 and NMS-873 resistant cells (Figure S3E, Supplementary Materials), which indicates that the drugs are synergistic [50,51,52,53]. Taken together with the synergistic anti-proliferation effect and the difference of oxidative phosphorylation inhibition IC_50_ between normal and tumor cells, using 2-DG as an adjuvant agent may be an excellent approach for decreasing the dosage of NMS-873, reducing possible lactic acidosis, and relieving NMS-873-induced resistance. Our studies provide a deeper understanding of NMS-873 as well as new approaches to potentially optimize the potency of NMS-873 as an anti-cancer agent potentially through polypharmacology effects [60,61,62] as shown in Figure 6.

## 5. Conclusions

In conclusion, we discovered that NMS-873 dysregulates glycometabolism in a p97-independent manner and affects the stability of mitochondrial complex I in HCT116 cells by combining generation of resistant cells, global proteomics analysis, and PISA-T approaches. We found that 2-DG has a synergistic anti-proliferative effect with NMS-873 and this serve as a potential combination therapy for colon cancers. The polypharmacology effects of NMS-873 can be a potential usefulness of a compound that can hit multiple important protein targets in colon cancer cells.

## Data Availability

The mass spectrometry proteomics data have been deposited to the ProteomeXchange Consortium via the PRIDE [63] partner repository with the dataset identifier PXD025898 and 10.6019/PXD025898. All relevant data generated during this study are included in the article and the Supplementary Information. This paper does not report original code. Any additional information required to reanalyze the data reported in this paper is available from the lead contact upon request.

## Supplementary Materials

The following supporting information can be downloaded at: https://www.mdpi.com/article/10.3390/pharmaceutics14040764/s1. Figure S1: Additional data of NMS-873 regulates glycometabolism; Figure S2: Additional data of generating NMS-873 resistant cell lines; Figure S3: More data to prove NMS-873 affects glycometabolism in a p97 independent manner, and synergistic anti-proliferative effect of 2-DG and NMS-873; Figure S4: Proteomic analysis of 4 μM NMS-873-treated HCT116 and NMS-R2 cell lines; Figure S5: Volcano plot showing DS proteins of a repeated PISA T assay; Figure S6: of Additional PISA T proteomic data and NDUFAF5-HA expression result. Table S1: DE proteins of p97 inhibitors and MG132 treatments, related to Figure 1A,B; Table S2. Proteomic data of NMS-873 treated HCT116 and NMS-R2, related to Figure 3E and Figure S4; Table S3. Proteomic data of the PISA T assay using crude cell extracts and one temperature range, related to Figure S5; Table S4. Proteomic data of the PISA T assay using cell lysate and two temperature ranges, related to Figure 4A–D and Figure S6A; Table S5. Functional enrichment analysis of DS proteins from PISA T assay, related to Figure 4E and Figure S6B; Table S6. Mitochondrial complexes DS proteins from PISA T assay, related to Figure 4F; Table S7. DS proteins identified in both PISA T assays, related to Figure 4B,C and Figure S5; Table S8. PCR and sequencing primers used in the study.
