# Supplementary material for: NMS-873 Leads to Dysfunctional Glycometabolism in A p97-Independent Manner in HCT116 Colon Cancer Cells
Source: Pharmaceutics. 2022 Mar 31;14(4):764. doi: 10.3390/pharmaceutics14040764 (PMC9024726; doi:10.3390/pharmaceutics14040764)
Supplement: Supplementary file 1 [file pharmaceutics-14-00764-s001.zip › pharmaceutics-1631960 SM.pdf]

Supplementary Materials

NMS-873 Leads to Dysfunctional Glycometabolism in a p97-Independent Manner in HCT116 Colon Cancer Cells

Shan Li, Feng Wang, Gang Zhang, and Tsui-Fen Chou

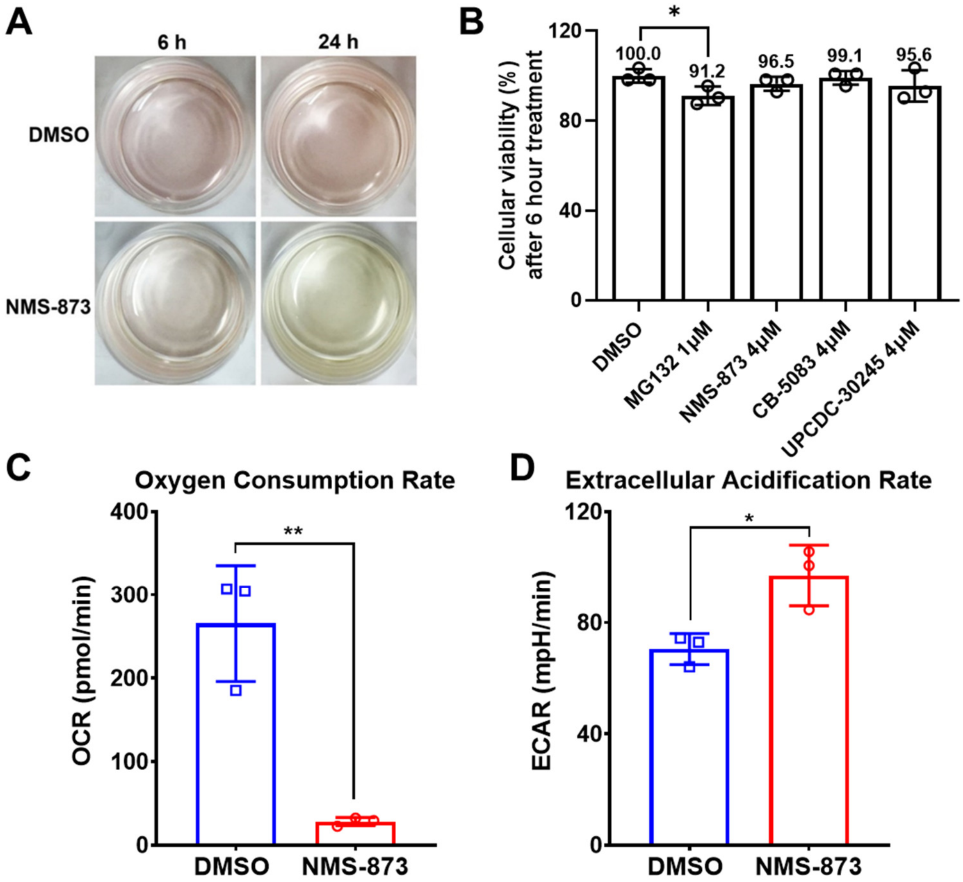

Figure S1. (**A**) HCT116 cell culture dishes with DMSO or 4 μM NMS-873 for 6 and 24 hours. (**B**) Cell viabilities of HCT116 cells after 6 hour treatment of DMSO or compounds. (**C,D**) HCT116 cells were treated with DMSO or 2 μM NMS-873 for 6 hours; oxygen consumption rate (OCR) **(C)** and extracellular acidification rate (ECAR) **(D)** were measured using the Seahorse instrument. n = 3, statistical analyses were carried out by *t*-test using Prism 8. P-values are shown as * $p < 0.05$, ** $p < 0.01$.

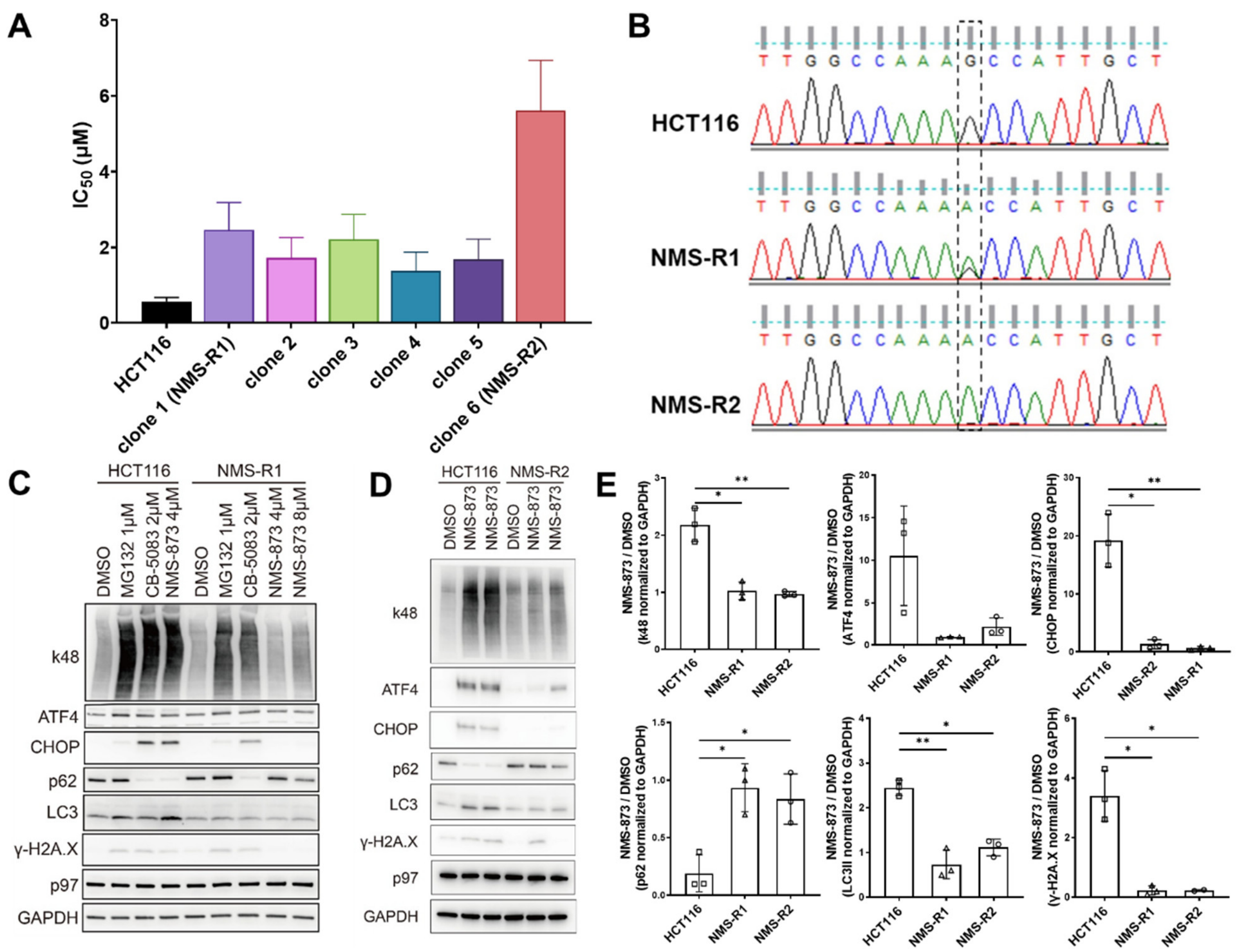

Figure S2. (A) Anti-proliferation activity of NMS-873 against HCT116 and six isolated resistant clones. **(B)** Sequencing results of cDNA from HCT116 and NMS-873 resistant cell lines. **(C,D)** Western blot results of HCT116 and NMS-R cell lines. **(E)** Quantification of p97 biomarkers from western blot. n = 3, statistical analyses were carried out by paired *t*-test, ** indicates $p < 0.01$, * indicates $p < 0.05$.

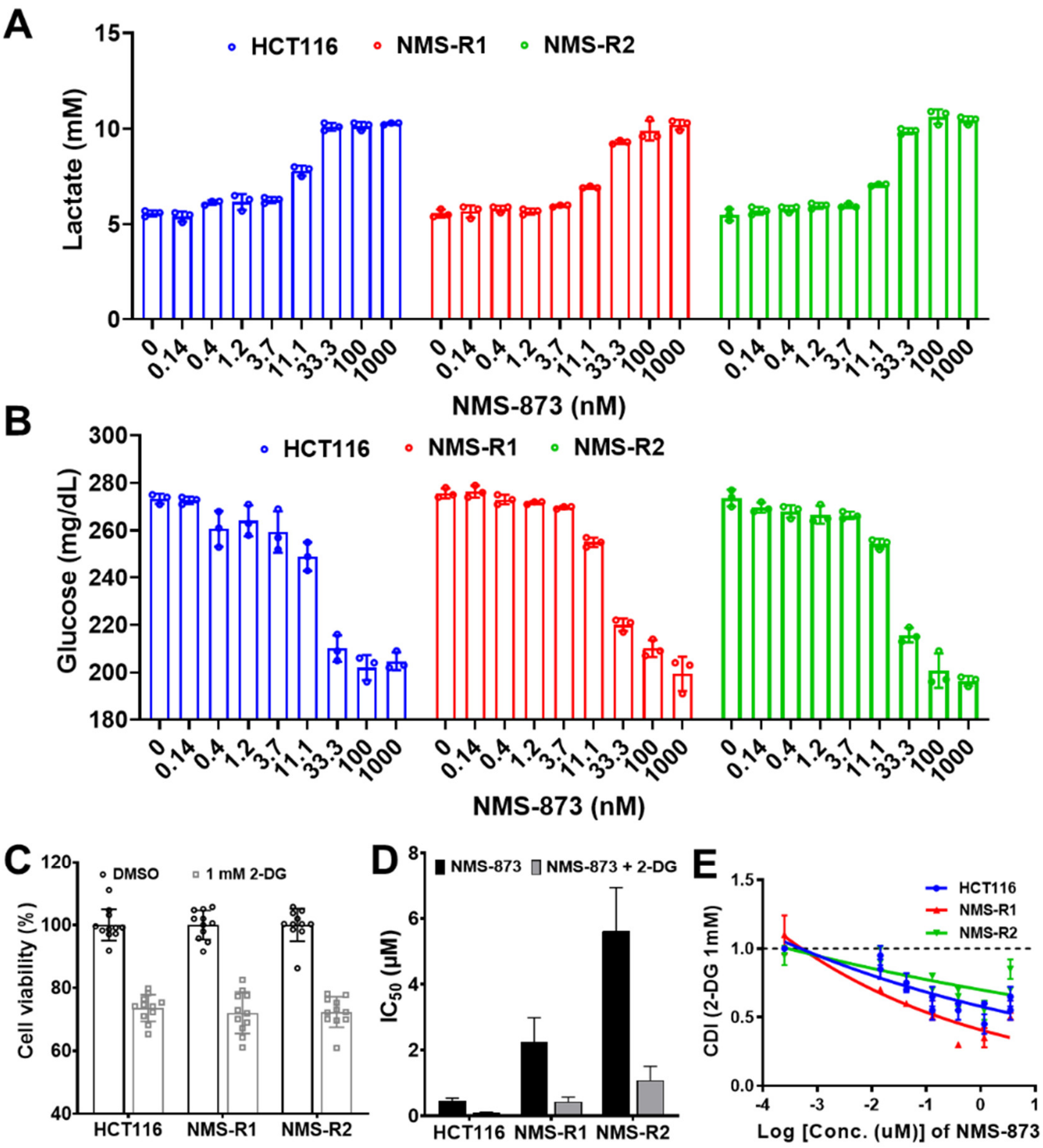

Figure S3. Lactate production **(A)** and glucose consumption **(B)** were measured in the medium of parental and NMS-873-resistant cells after being treated with different concentrations of NMS-873 for 6 hours. Data are presented as mean ± SD from triplicate experiments. **(C)** Effects of 1 mM 2-DG on cell viability. **(D)** Anti-proliferation activities of NMS-873 alone, treated and co-treated with 1mM 2-DG against HCT116 and resistant cells. Data are presented as mean ± SD from quadruplicate experiments. **(E)** The coefficient of drug interaction (CDI) of different doses of NMS-873 and 1 mM of 2-DG mixtures on their anti-proliferation effect in HCT116 and NMS-873 resistant cells. CDI is calculated as follows: CDI = AB / (A X B). AB is the ratio of the combination groups to control group; A or B is the ratio of the single agent group to control group, n = 4.

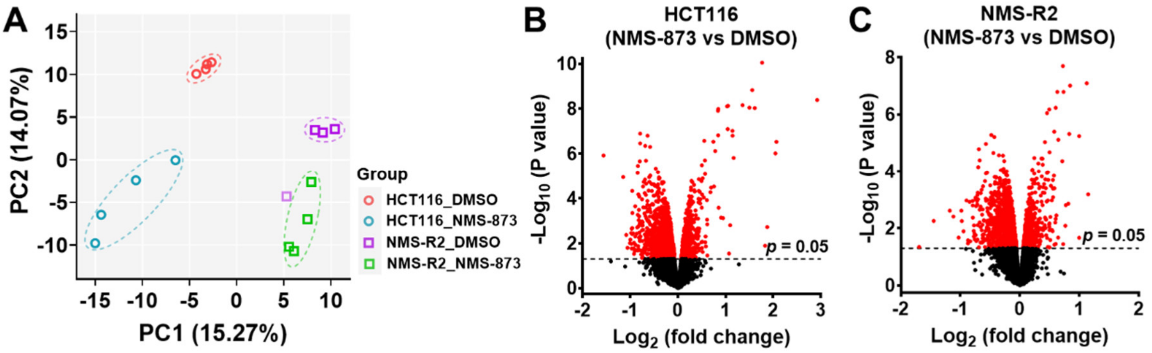

Figure S4. Proteomic analysis of 4 μM NMS-873-treated HCT116 and NMS-R2 cell lines (n = 4). **(A)** Principal component analysis (PCA) displaying the separation of different groups. **(B-C)** Volcano plots showing DE proteins following NMS-873 treatment in HCT116 and NMS-R2 cells. DE proteins were defined as treatment vs. DMSO control, $p < 0.05$.

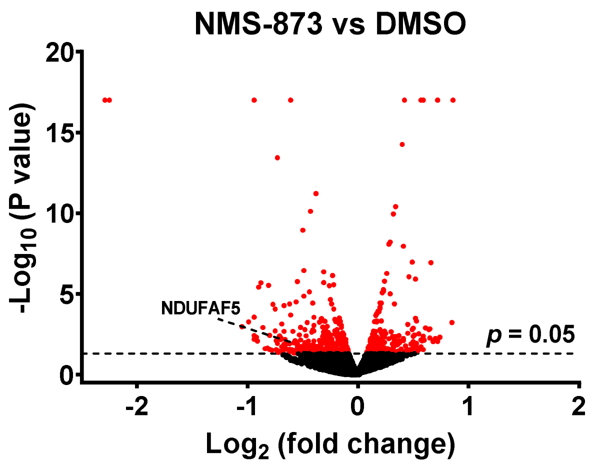

Figure S5. Volcano plot showing DS proteins of a repeated PISA T assay using crude cell extracts from 4 freeze-thaw cycles and combining samples of the whole temperature range (n = 3). DS proteins were defined as treatment vs. DMSO control, $p < 0.05$. (The list of proteins is shown in Table S3).

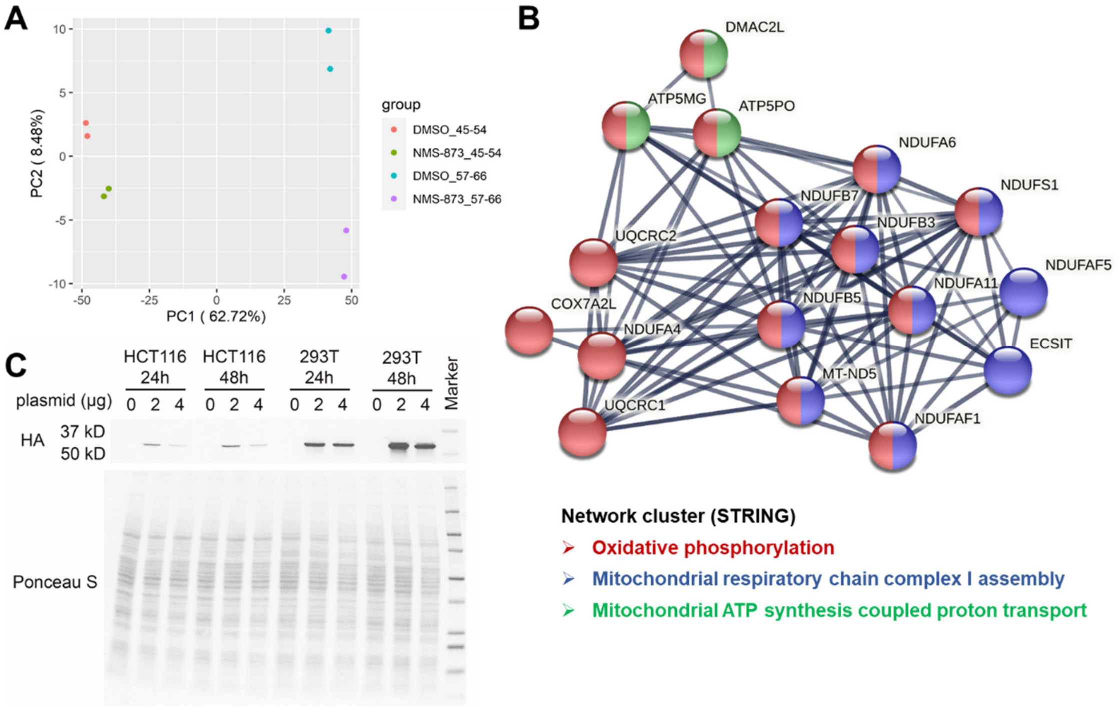

Figure S6. (A) PCA analysis of PISA T proteomic data showed the separation of each treatment. **(B)** Protein-protein interaction network of DS proteins listed in glycometabolism-related GO biological processes by STRING. The minimum required interaction score was set at high confidence (0.700). **(C)** NDUFAF5-HA expression level test by transiently transfecting different amounts of the plasmid to HCT116 or 293T cells for 24 or 48 hours.

Table S1. DE proteins of p97 inhibitors and MG132 treatments, related to Figure 1A and 1B

Table S2. Proteomic data of NMS-873 treated HCT116 and NMS-R2, related to Figure 3E and S4

Table S3. Proteomic data of the PISA T assay using crude cell extracts and one temperature range, related to Figure S5

Table S4. Proteomic data of the PISA T assay using cell lysate and two temperature ranges, related to Figure 4A–D and S6A

Table S5. Functional enrichment analysis of DS proteins from PISA T assay, related to Figure 4E and S6B

Table S6. Mitochondrial complexes DS proteins from PISA T assay, related to Figure 4F

Table S7. DS proteins identified in both PISA T assays, related to Figure 4B,C and S5

Table S8. PCR and sequencing primers used in the study.

Name of primer	Sequence of primer
VCP-Cloning R	5′- ACC CCC AGG GAA CAA G -3′
VCP-Cloning F	5′- CAG CGT TGT TCG CCC -3′
p97 bp301F	5′- GCA TCC AGC CAT GCC CTG ATG TG -3′
p97 bp803F	5′- GAC CCT GAT TGC TCG AGC TG -3′
p97 bp1189F	5′- GAA CAG GTA GCC AAT GAG ACT -3′
p97 bp1501F	5′- GAC AAA TTC CTG AAG TTT GGC -3′
p97 bp1976F	5′- CTA ACC TGC GCA AGT CCC CAG TTG -3′
